# Alternative Oral Agents in Prophylaxis and Therapy of Uterine Fibroids—An Up-to-Date Review

**DOI:** 10.3390/ijms18122586

**Published:** 2017-12-01

**Authors:** Michał Ciebiera, Krzysztof Łukaszuk, Błażej Męczekalski, Magdalena Ciebiera, Cezary Wojtyła, Aneta Słabuszewska-Jóźwiak, Grzegorz Jakiel

**Affiliations:** 1 Department of Obstetrics and Gynecology, The Centre of Postgraduate Medical Education, 00-416 Warsaw, Poland; czwo@op.pl (C.W.); as.jozwiak@op.pl (A.S.-J.); grzegorz.jakiel1@o2.pl (G.J.); 2 Department of Obstetrics and Gynecological Nursing, Faculty of Health Sciences, Medical University of Gdansk, 80-210 Gdansk, Poland; luka@gumed.edu.pl; 3 INVICTA Fertility and Reproductive Center, 80-172 Gdansk, Poland; 4 Department of Gynecological Endocrinology, Poznan University of Medical Sciences, 60-513 Poznan, Poland; blazejmeczekalski@yahoo.com; 5 Students’ Scientific Association at the I Department of Obstetrics and Gynecology, Medical University of Warsaw, 02-015 Warsaw, Poland; mciebiera93@gmail.com

**Keywords:** uterine fibroid, leiomyoma, vitamin D, paricalcitol, epigallocatechin gallate, elagolix, aromatase inhibitors, cabergoline, pharmacology

## Abstract

Uterine fibroids (UFs) are the most common tumors of the female genital tract. The effect of UFs on the quality of life and the overall cost of treatment are significant issues worldwide. Tumor size and location are the two specific factors which influence the occurrence of symptoms, the need for, and method of, treatment (some tumors require surgery while some can be treated with selected drugs). Primary prevention and treatment of early UF disease are worthy goals that might have a great impact on health care systems. Several treatments and prophylactic methods can be used in this endeavor. This publication presents current data about lesser-known substances which may have a beneficial effect on the treatment or prophylaxis of UFs and can be administered orally, serving as an alternative to (or complement of) surgery or selective progesterone receptor modulators (SPRMs). Early prevention and treatment of UFs in women from high-risk groups should be our priority. Innovative forms of UF management are under intensive investigation and may be promising options in the near future. Many of them evaluated vitamin D, paricalcitol, epigallocatechin gallate (EGCG), elagolix, aromatase inhibitors (AIs), and cabergoline and deemed them to be safe and effective. The next step in such projects should be properly constructed randomized control trials (RCTs), carried out by successive phases.

## 1. Introduction

Uterine fibroids (UFs)—benign monoclonal tumors protruding from myometrial smooth muscle cells—are the most common pathology of the female genital tract [1,2,3]. The morphology of UFs may vary greatly. They can be solitary or appear in multiple clusters. Also, their size range is considerable, too, from miniscule to giant masses of over 20 cm in diameter. UFs affect 25–80% women, depending on the population and the risk factors [2,4,5]. The majority of UFs are asymptomatic, and menopause generally results in tumor atrophy, but the symptomatic tumors constitute a major problem for the vast number of affected women. The wide range of UF-associated symptoms includes iron deficiency anemia, abdominal and pelvic pain, gastrointestinal disorders, dysuria, female infertility, and severe obstetric complications [2,4,6].

This pathology varies greatly in relation to age. These tumors are more prevalent among older populations [2,7,8]. UFs are not observed in pre-pubescent girls and are a rare finding in adolescents, indicating that they depend on hormonal changes [7,9]. According to the available data, the growth of UFs depends mostly on the influence of steroid hormones [1,10,11]. Estrogens have been known to play an important role in the pathophysiology of UFs, but the latest research has suggested progesterone as the main factor initiating pathological uterine muscle differentiation and abnormal growth [1]. The main mechanism of progesterone-induced UFs tumorigenesis consists of an increase in the concentration of selected growth factors [1,12,13]. Also, a significant part of UFs occurs due to a genetic abnormality [1]. In patients with positive family history, the risk for developing UFs approximately 4 times higher than in the general population [14]. According to a study by Makinen et al., specific mutations within the gene encoding the mediator complex subunit 12 (*MED12*) were detected in the examined UFs [15]. Nowadays, it is a known fact that even up to 80% of UFs have a mutation in *MED12* [1,15,16]. 

UFs are a major public health problem. By the age of 50, they might develop in almost 80% and 70% of the African-American and the Caucasian women, respectively [3,8]. The effects of UFs on the quality of life (QoL) and the overall cost of treatment are significant but often remain unaddressed or marginalized [17]. As far as QoL for women in general is concerned, Soliman et al. have recently demonstrated that women who rated their UF-related symptoms as “severe” had significantly worse QoL as compared to their peers with mild symptoms [18]. QoL deteriorated considerably with the increasing number and severity of symptoms [18]. A 2015 review of the literature on direct and indirect costs of UF management revealed that substantial sums of money are generated by UFs [19], and included not only the price of medicines, medical staff salaries, or the cost of surgical treatment, but also the hidden costs of work absence, hospitalization, control visits, and preoperative diagnostic tests. The annual direct and indirect costs of UFs in the United States have been estimated to be between $4.1–$9.4 billion [3,19,20], and $1.6–$17.2 billion, respectively [20]. In the United States, the total cost of treatment of a single patient with UFs ranges from $11,700 to $25,000 per year after the diagnosis or surgery [19,21]. According to a well-known study by Cardozo et al., the total annual cost of UF treatment in the United States has been estimated at $34.4 billion [20]. 

Tumor size and location determine the occurrence of symptoms, the need for treatment, and the treatment method. Other important determinants include symptom severity, patient age and reproductive plans, the risk for malignancy, skills and expertise of the gynecologists and access to proper medical equipment [2,6]. Due to the benign nature of UFs, treatment resulting in the least morbidity and lowest risk should be chosen, if possible [2,6,22]. Multiple UF management options are currently available but surgery remains the method of choice and is often accompanied by pharmacological treatment or pretreatment [2,22,23,24]. The most common complaint—menorrhagia—is managed with surgical procedures like ablation, myomectomy or uterine artery embolization or, more recently, by pharmacotherapy [6,22]. The available treatments for UFs, including hysterectomy, myomectomy, embolization, and gonadotropin-releasing hormone (GnRH) agonists, are effective but are recommended in more advanced stages of the disease, especially since they are neither low-cost nor free of risk for adverse events [25,26]. Ulipristal acetate (UPA), a selective progesterone receptor modulator (SPRM), is the most common UF pharmacological treatment [6,22,26,27,28]. Clinical trials have demonstrated that UPA is effective for controlling UF-related excessive uterine bleeding and reducing fibroid size [6,22,26,27]. Treatment schemes with UPA have recently become the gold standard in modern management of UFs [27]. In those schemes, UPA is administered as first-line therapy to prepare UFs for surgery or, in case of good response, to lead to a condition when surgical treatment is no longer necessary [6,29]. However, UPA is relatively expensive and not accessible to everyone, nor is it a substance which can be widely used in prevention [2,22]. In spite of the ongoing research, the currently available pharmacological therapies are short-term, to avoid the risk of chronic hormonal therapy, and are accompanied by long-term adverse side effects.

One of the major problems associated with UFs is that they are understudied, and therefore, much research is needed in this field [25,30]. The development of UFs is multi-factorial in origin, thus specific methods of prophylaxis are currently unavailable. Various recent attempts to create an inexpensive, safe, and effective drug for the prevention and treatment of UFs are still in the early stages of the process [31]. Still, several treatments and prophylactic methods can be used in this endeavor [31]. Current findings suggest that substances contained in green tea [32], vitamin D [25,33], elagolix [34], paricalcitol [35], gestrinone [36], and others may become future preparations for chronic treatment with minimal or moderate side effects. Similar concepts are discussed in an attempt to create a specific method of prophylaxis of UFs in high-risk subjects [14,25,37]. High costs and low efficacy are the key points in UF therapy. If more such substances are known, it will be possible to investigate them further (new dosages and schemes), and combine their effect with the known agents to achieve better performance. This, in turn, may have a great impact upon the health of millions of women in the future.

This publication presents current data about lesser-known substances which may have a beneficial effect on the treatment or prophylaxis of UFs and can be administered orally, serving as an alternative to (or complement) surgery or SPRMs.

## 2. Materials and Methods

This article presents an up-to-date review of publications regarding the current role of alternative agents in UF treatment and prophylaxis. A literature search was conducted in PubMed of the National Library of Medicine using the following key words: “uterine fibroid”, “pharmacotherapy”, “vitamin D”, “vitamin D analog”, “paricalcitol”, “gestrinone”, “elagolix”, “aromatase inhibitor”, “epigallocatechin gallate”, “green tea”, “curcumin”, and “cabergoline”. The above keywords were selected to reflect possible oral agents in the prophylaxis and therapy of UFs. During our search, we combined the key words into pairs, which resulted in: “uterine fibroid” and “pharmacotherapy”—1694 publications; “uterine fibroid” and “vitamin D”—40 publications; “uterine fibroid” and “vitamin D analog”—2 publications; “uterine fibroid” and “paricalcitol”—2 publications; “uterine fibroid” and “gestrinone”—19 publications; “uterine fibroid” and “elagolix”—1 publication; “uterine fibroid” and “aromatase inhibitor”—73 publications; “uterine fibroid” and “epigallocatechin gallate”—7 publications; “uterine fibroid” and “green tea”—8 publications; “uterine fibroid” and “curcumin”—4 publications; “uterine fibroid” and “cabergoline”—4 publications. If the search was duplicated the papers were excluded. The aim of the review was to critically evaluate the current data about lesser-known substances which might have a serious impact on UFs and UF-related symptoms and which can be administered orally. The results of the available studies in English, published up to October 2017, have been discussed in this article. Additional important and impactful articles and reviews were considered, when relevant. Articles were excluded if they were published in languages other than English. After reviewing the titles and abstracts, approximately 150 full articles have been evaluated.

## 3. Discussion

### 3.1. Vitamin D

The least studied factors which affect the risk for UF occurrence are related to lifestyle, diet, nutrition, or place of residence. This can be the gateway to effective prevention of UFs. Vitamin D is the part of the fat-soluble steroid compound group which exerts comprehensive action on the human body [38]. Sunlight exposure is the main source of vitamin D for humans [39]. This vitamin can also be extracted from diet or food supplements. However, very few natural foods contain vitamin D in the appropriate amount. Marine fish, fish oils, and fortified food are among the best sources of vitamin D [40].

Vitamin D is believed to reduce the risk for chronic illnesses and malignancies [38,41,42], and has a potent immunomodulatory function. Vitamin D receptor (VDR) is expressed in almost all cells of the immune system where their function is regulated [43,44]. Vitamin D regulates cell proliferation and differentiation, inhibits angiogenesis, and stimulates apoptosis [33,45]. Vitamin D deficiency is believed to be a major risk factor in the development of UFs [14,33,37,46]. According to the recent literature reports, mean 25-hydroxyvitamin D (25(OH)D) levels are significantly lower in women with UFs as compared to controls [47,48]. The same was confirmed in African-Americans, who are more likely to present vitamin D deficiency and UFs [49]. Three main studies on reduced serum vitamin D levels in women with UFs have revolutionized the approach in this field and have unambiguously targeted research for the upcoming years [46,50,51].

**Figure 1:**
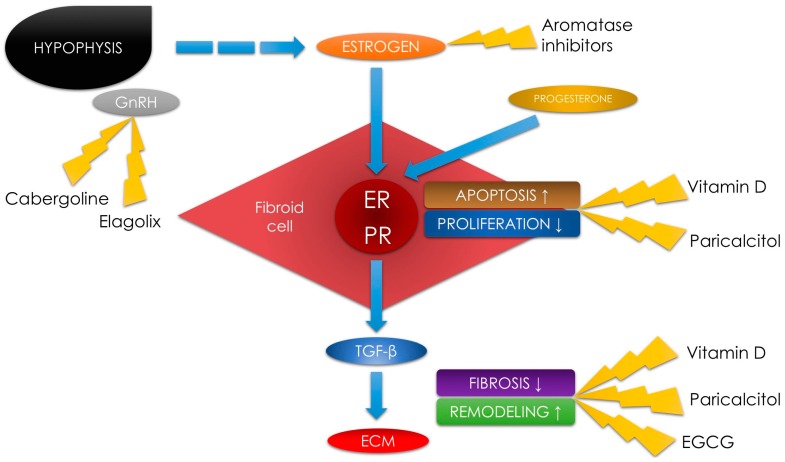
Schematic diagram of uterine fibroid pathophysiology with places for potential use of an alternative agent therapy. Gonadotropin-releasing hormone (GnRH), estrogen receptor (ER), progesterone receptor (PR), transforming growth factor beta (TGF-β), extracellular matrix (ECM), epigallocatechin gallate (EGCG).

New research revealed that a protective effect of vitamin D on UFs is highly likely. VDR is expressed in both, myometrial and tumor tissues [52]. A correlation between low 25(OH)D serum levels and as increased risk for developing UFs was evaluated [14,33,46,51]. According to in vitro experiments, UF cells are highly sensitive to the growth-inhibiting effect of the active form of vitamin D. In those studies, vitamin D induced apoptosis in cultured human UF cells through the downregulation of proliferating cell nuclear antigen (PCNA), cyclin-dependent kinase 1 (CDK1), and B-cell lymphoma-2 (BCL2) and suppressed catechol-*O*-methyltransferase (COMT) expression and activity [31,52,53,54] (Figure 1).

In a study by Halder et al., UFs in rats have diminished in size under the influence of this vitamin on the transforming growth factor beta 3 (TGF-β3) pathway, as vitamin D reduces the TGF-β3-induced fibrosis [55]. In a different study, Halder et al. proved that administration of 1,25-dihydroxyvitamin D reduced the expression of type I collagen and fibronectin in UFs [56]. In our recent study, we also found that vitamin D might play a role in decreasing TGF-β3 levels in women [14,21] (Figure 1).

Vitamin D toxicity is very rare [41]. The recommended level of vitamin D is defined as a 25(OH)D value of more than 30 ng/mL [57]. According to the summarized supplementation guidelines developed in 2017 (7000 international units (IU)/day or 50,000 IU/week), deficiency may be remedied by adequate supplementation [58]. Vitamin D supplementation and sunlight exposure are the two main measure for prevention of a wide spectrum of disorders, including UFs [59,60].

Our study emphasizes the need for new clinical trials to assess the actual effectiveness of vitamin D in UF therapy. The next step should be a properly constructed, randomized clinical trial. Vitamin D seems to be a promising, safe, effective, and low-cost treatment of UFs. In cases of positive observations, vitamin D preparations could become a new generation of anti-UF drugs, with the additional beneficial pleiotropic effect. Additional skeletal (bones and ligaments) and extra-skeletal (other organs and overall homeostasis) advantages support the use of vitamin D as a prophylactic agent [25,38,58]. Further data are needed to fully comprehend the exact role of vitamin D in the pathophysiology of UF. To the best of our knowledge, there are still no randomized controlled trials (RCTs) on this subject, the main reason for that being lack of on the cut-off thresholds for vitamin D deficiency, optimal levels and dosage. However, it seems that the near future will finally bring the consensus [58].

### 3.2. Vitamin D Analogs—Paricalcitol

Due to the potentially adverse or even toxic effects of vitamin D in very high concentrations, consideration should be given to possible alternatives [35]. Vitamin D analogs are already present on the pharmaceutical market and their possible beneficial effects are intriguing. Paricalcitol (a well-known vitamin D analog) is a selective VDR activator which is registered and used in secondary hyperparathyroidism [61]. According to Bouillon et al., paricalcitol has less calcemic activity than vitamin D and might be a new option in modern therapy [62]. Paricalcitol can cause electrolyte abnormalities like hypercalcemia and hyperphosphatemia, and is contraindicated in patients who use digoxin, thiazide diuretics, and ketoconazole [63]. Paricalcitol has a proven inhibitory effect on cell proliferation and fibrosis [35,64]. It has also been found to reduce the increase of the extracellular matrix (ECM) accumulation in the peritoneum of dialyzed patients [65]. In a study by Stavenuiter et al., the authors concluded that the immunomodulatory effects of paricalcitol have beneficially contributed to the development of ECM (limiting its thickening), and that VDR activation can partly increase the kidney filtration rate due to limitation of angiogenesis and thickening of ECM [65].

Vitamin D reduces inflammation and fibrosis by VDR activation [66,67]. According to a study by Protic et al., cytokines play a key role in inflammation and tissue remodeling regulation, indicating that they may be responsible for UF-associated symptoms such as pain or infertility [45]. TGF-β (mainly TGF-β3 isoform) plays one of the major roles in UF-related fibrosis [14,54,68,69]. In their study, Oblak et al. demonstrated a significant decrease in TGF-β serum concentrations in transplant recipients who received paricalcitol as compared to controls [66]. Another study by Gonzales-Mateo et al., showed that the use of paricalcitol strongly reduced peritoneal interleukin (IL)-17 levels, which might correlate with lower peritoneal membrane deterioration and lower fibrotic response in the peritoneum in mice models [44]. As for the relevance of the abovementioned studies to UF therapy, UFs consist largely of ECM with embedded cells and excessive ECM production is considered to be one of the mechanisms of UF formation [70]. The ECM which builds the uterus is much more abundant than properly functioning myometrial tissue [71]. Matrix metalloproteinases (MMPs) are calcium-dependent endopeptidases which degrade the structure and rebuild the ECM [72]. ECM undergoes a continuous and balanced reconstruction process which contributes to the maintenance of its proper amount and hardness. Matrix enzymes are regulated by special inhibitors of ECM metalloproteinases (tissue inhibitor of metalloproteinases (TIMP)) [73] (Figure 1). Recent research has demonstrated that vitamin D increased TIMP expression in uterine myometrium [56]. 

In conclusion, paricalcitol effectively reduces the proliferation of human leiomyoma cell cultures, reduces fibroid tumor volumes, and induces apoptosis in UFs [35]. In our opinion, paricalcitol may be an effective agent in UF therapy. It has great potential as an effective drug or co-drug for the conservative treatment of UFs. However, advanced clinical trials are necessary to confirm its efficacy and safety [35]. To the best of our knowledge, there have been no RCT on the use of paricalcitol in UF therapy.

### 3.3. Green Tea Extract—Epigallocatechin Gallate

Tea is one of the most popular beverages in the world. Green tea is made from Camellia sinensis leaves [74,75]. It originated in China, but later spread to many parts of Asia. Green tea contains several catechins with treatment potential: epigallocatechin gallate (EGCG), epigallocatechin (EGC), epicatechin gallate (ECG), epicatechins, and flavonols [76].

Various researches suggested that polyphenols have antioxidative, anticarcinogenic, and anti-inflammatory effects in humans [75,77]. Currently, EGCG, an ester of epigallocatechin and gallic acid, is one of the most investigated green tea polyphenols in medicine. The effect of EGCG was studied in oncology, where it presented beneficial effects [78,79]. EGCG is an anti-obesity and anti-adipogenic agent due to its activity in the adipogenic differentiation of mice mesenchymal stem cell inhibition [80]. One of the key pathways in this process is based on the gamma protein blocking activity of EGCG human peroxisome proliferator activated receptor gamma (PPARγ) [80]. In non-acute fatty liver disease animal models, EGCG reduced the concentration of pro-fibrotic and pro-inflammatory factors in several different pathways [81]. In studies on pulmonary fibrosis, EGCG significantly inhibited fibroblast activation and ECM accumulation by blocking the TGF-β1 signaling pathway [82].

The question arises whether adequate amounts of green tea might exhibit protective effect against UFs. In an interesting study by Matsuzaki et al., EGCG was found to inhibit proliferation and invasion of endometrial implants in animals [83]. In molecular findings, EGCG reduced the TGF-β-dependent mRNA expression of fibrosis mediators [83] (Figure 1). These authors concluded that EGCG might be a potential candidate in the treatment of endometriosis [83]. The only problem with this agent is its low bioavailability in natural sources [83,84,85], but several studies regarding the improved derivatives, analogs, or prodrugs of EGCG have demonstrated positive and promising results [86,87].

The effectiveness of EGCG has been studied in UF pathophysiology, and the results are more than interesting [72]. Some authors believe that it might be a new weapon in the battle against UFs [22,88]. EGCG was found to inhibit proliferation and reduce the volume of UF tumors in mice [89]. Various studies have demonstrated its effect on inhibition of proliferation and induction of apoptosis in human UF cells [32]. In those studies, EGCG was a modulation agent in the proliferation, transformation, and inflammation in UF tumors [32,50]. The pathophysiological pathways proving EGCG modulation potential included PCNA, CDK1 and BCL2 (same as in vitamin D) [31]. Studies by Zhang et al., demonstrated that UFs in mice and human UF cell colonies were shrinking under the influence of EGCG [32,89] (Figure 1). Roshdy et al. evaluated the oral use of EGCG in women with symptomatic UFs and the results are highly encouraging. Women who received 800 mg of 45% EGCG for 4 months had a significant reduction of the total fibroid tumor burden and alleviation of symptom severity as compared to controls [88]. More recently, Ahmed et al. presented interesting data about potent prodrugs and analogs of EGCG which resulted in enhanced bioavailability, stability, and antiproliferative and antifibrotic properties. These findings enable us to discern that green tea extract might have found its place in UF management. Those authors are planning broader studies regarding these drugs which, in our opinion, might be revolutionary [87]. If the results about long-term safety and efficacy are positive, EGCG might become the new quality treatment in UF management [87]. As EGCG is now available as a dietary supplement [90], it would also be interesting to investigate the possible effects of combining it with vitamin D or paricalcitol.

The literature lack data on safe dose levels of pure EGCG. According to the available sources, hepatotoxicity was observed in animal studies with the use of high doses of EGCG. Ramachandran estimated the maximum tolerable dose of EGCG at 67.8 mg/kg orally (in the course of two weeks) [91].

In our opinion, available data suggest that EGCG has a very high potential to become an alternative agent in the prophylaxis or anti-UF therapy, but RCTs on this subject are unavailable.

### 3.4. Elagolix

GnRH agonists are highly effective drugs in the management of symptomatic endometriosis [92,93], as well as in UFs [2,6]. Alas, they can cause severe hypoestrogenic effects, such as vasomotor symptoms (e.g., hot flashes), which will limit the treatment duration or cause the patient to discontinue the therapy due to the reduced QoL [93]. The treatment of benign gynecological diseases does not require full estrogen suppression to partly or even fully reduce the symptoms [93].

Elagolix is a potent and selective non-peptide antagonist of the GnRH receptor [94,95]. It is one of very few (with relugolix [96], and OBE2109 [97]) GnRH antagonists which can be administered orally, and its administration in proper doses suppresses the reproductive endocrine axis in healthy premenopausal women [98]. According to several studies, elagolix is superior to the currently available GnRH agonists and antagonists due to its manageability, rapid onset of action, good bioavailability, rapid reversibility, and minor side effects [93,98]. Very recently Ng et al. published their data supporting elagolix administration in premenopausal women with sex-hormone dependent diseases [93]. In their opinion, elagolix might be useful in modulating the pituitary–ovarian axis by dose-dependent partial or full suppression [93] (Figure 1). Studies about the intense pain associated with endometriosis demonstrated that elagolix has good efficacy and tolerability. Very recently, Taylor et al. studied 872 women and found that elagolix has great potential as an important treatment option for women suffering from endometriosis. In this study, women who received elagolix had significantly lower scores for dysmenorrhea and non-menstrual pelvic pain than placebo controls [99].

Even if its activity and influence have been tested mostly in endometriosis [95,99], there are also studies about the use of elagolix in UF management. Recently, Archer et al. presented their results regarding different doses of elagolix in heavy menstrual bleeding [34]. Elagolix reduced heavy menstrual bleeding symptoms in women with UFs. Optimal results were obtained using the dose of 300 mg twice per day [34]. These authors observed hypoestrogenic side effects such hot flashes (most frequent), nausea, or headache in some patients, but they were significantly reduced by the use of low-dose estrogen add-back therapy [34]. They concluded that elagolix is a candidate for becoming the new medication in the chronic treatment of clinically symptomatic UFs and should be moved to the next phase of the clinical trials [34]. Phase III studies determined that elagolix achieves its primary endpoints in endometriosis, while adverse events during its use were described as mild to moderate (the most common ones are headache, nausea and anxiety) [100]. Data about its toxicity remains limited.

The pharmaceutical company currently undertaking elagolix clinical trials intends to submit elagolix as a drug in endometriosis therapy to the United States Food and Drug Administration in 2017 [101]. Phase III clinical trials of elagolix in UF therapy are underway [101]. Even if this new GnRH antagonist is well tolerated, much remains to be discovered. We need further studies in larger numbers for longer time periods to gain additional information about the efficacy, tolerability, and compliance of elagolix [34,100].

### 3.5. Aromatase Inhibitors

Aromatase is one of the major enzymes responsible for estrogen synthesis. It can be found in various tissues, including gonads, adipocytes, blood vessels, and the nervous, integumentary, and skeletal systems, as well as endometrial implants, UF tumors, or gynecological cancers [102,103]. Aromatase inhibitors (AIs) decrease the production of estrogens by blocking or inactivating aromatase. AIs are a class of drugs which demonstrate the antiestrogenic effect (Figure 1). The most well-known AIs are anastrozole, letrozole, and fadrozole. They are mainly used in the treatment of gynecological cancers (breast or endometrial cancer) [104], to suppress estrogen production [104], and to treat endometriosis [105,106,107] or infertility (induction of the ovulation) [107]. AIs have good efficacy, but they can cause common side effects, such hot flashes, bone loss, mood swings, vaginal dryness, ovarian cyst formation, or body pain [108]. The use of AIs in premenopausal women can rise the plasma estrogens levels by the stimulation of gonadotropins [109,110]. In peri- or post-menopausal women, estrogens are mainly produced in the peripheral tissues (e.g., adipocytes). Using AIs in this patient group would decrease estrogen levels in both, plasma and tissues [109,110]. Postmenopausal women are already exposed to a low-hormone environment, and the use of AIs can lead to better results in the treatment of hormone-dependent diseases [111].

There are several studies about the effect of AIs on UF and UF-related symptoms [108]. Notably, UF tissue expresses aromatase in higher amounts than normal myometrial tissue [112], and aromatase has decreased expression in Japanese and Caucasian subjects as compared to African-Americans, who have a higher rate of UFs [113]. On the basis of these findings, we can expect better results with the use of AIs in African-American populations [114]. Anastrozole has been described as an agent which shrinks UF tumors and reduces the clinical symptoms in post- [115] and peri-menopausal women without serious adverse events [116]. AIs were also found to have an advantage in the rapid onset of action [115,116]. In the study by Varelas et al., 3 month-use of anastrazole reduced mean UF volume by almost two-thirds, and these authors concluded that the use of AIs should be proposed to all women who want to avoid risky surgery [117]. In 2003, Shozu et al. published studies where AIs were shown to reduce UF size even up to 70% in just a 2-month period, with fewer side effects than GnRH analog therapy [22,118]. In an RCT comparing the effect of letrozole and triptorelin by Parsanezhad et al., letrozole presented better uterine volume reduction than the GnRH analog (45.6% vs. 33.2%, respectively) [109]. In this study, approximately 96% of patients in the triptorelin group reported related vasomotor symptoms, whereas in the AI group it was a rare finding [109]. In their review article, Shozu et al. concluded that certain doses of AIs can fully block the estrogen production in UF tumors, whereas ovarian production of estrogen would continue, but at reduced levels [118]. In this situation, UF tumors would shrink, and estrogen deficiency symptoms would be mild and tolerable [118]. In the case of concomitant symptoms during AI therapy, some authors advise the use of add-back treatment of GnRH analogs, estrogens, or progestins to prevent the increase in gonadotropin secretion and related symptoms [119].

A Cochrane review by Song et al., failed to present significant data which would support a wider clinical use of AIs in UF-related bleeding [120]. In 2013, reviewers concluded that the evidence of AI effectiveness and safety was insufficient to allow any conclusions to be drawn. In recent years, more studies about the role of AIs in the management of UFs have been published, and we hope that future systematic reviews and meta-analysis will gave us more answers about the use of AI in UF therapy [120]. In a study conducted by Duhan et al., a 12-week letrozole therapy reduced the mean UF volume by 52.5% in premenopausal women with clinically symptomatic tumors [121]. What is even more interesting, the side effects were mild (mostly hot flashes), and no significant effects were observed on lipid profiles and steroid serum levels [121]. Similar results were obtained by Sayyah-Melli et al. [122], who mixed letrozole with cabergoline. In their study, 12 weeks of treatment with letrozole with and without cabergoline improved the size and volume of UFs without significant differences between the study groups. Both groups were comparable for the remaining minor side effects [122]. In an interesting study from 2014, administration of letrozole and norethindrone acetate in patients with large UFs decreased the mean operative time by 13 min, intraoperative blood loss by 190 mL, and suturing time by 10 min during laparoscopic myomectomy as compared to placebo controls [123]. More recently, in another Italian study, the authors compared preoperative administration of triptorelin, letrozole, and UPA [124]. All medications caused a significant reduction in UF tumor volume, but the highest percentages were observed in the triptorelin and letrozole groups. The use of triptorelin and letrozole significantly decreased the mean hysteroscopy time and absorbed fluid, whereas these variables were insignificant in UPA group and controls [124].

Data about AI use in UF treatment are incomplete and demonstrate the need for more studies using larger sample size, longer time periods, and different doses. These studies will further our knowledge about the potential use of AIs in the treatment and prophylaxis of uterine leiomyomas. As estrogenic pathways are complicated and complex, patients may respond differently. We need to improve the efficacy or safety, perhaps by individualizing the doses and add-backs [9]. In recent studies, the AIs presented themselves as potent drugs in UF management which reduced UF volume and improved associated symptoms. Yet, due to limited data and lack of blinded trials, the use of AIs in prevention or early therapy of UFs remains debatable.

### 3.6. Cabergoline

Cabergoline is an ergot derivative and is a potent dopamine receptor agonist. Its overdose might cause nasal congestion, syncope, or hallucinations [125]. Cabergoline has an inhibitory effect on the secretion of GnRH, which may be the basis of its anti-UF effect [114,126] (Figure 1). Clinical data regarding the use of cabergoline in UF therapy are very limited. In a study by Sayyah-Melli et al., 0.5 mg of cabergoline per week had the same effect as 3.75 mg of diphereline (GnRH analog) per month on the reduction of UF volume [127]. Additionally, cabergoline presented a safer pharmacological profile, with fewer adverse drug reactions [127]. In another study, also conducted by Sayyah-Melli, cabergoline was added as a co-drug to letrozole to evaluate its synergic effect in UF therapy [122]. In this study, the addition of cabergoline did not significantly change the drop in UF volume, but caused more women to report a headache as an adverse reaction [101]. Finally, an Iranian study on 51 women showed that administration of cabergoline significantly reduced UF-related symptoms. In this study, women who received 0.5 mg of cabergoline daily for 3 months had decreased pelvic pain, reduced bleeding, and a lower volume of UFs as compared to controls [128].

According to abovementioned data, cabergoline might find its place in the treatment of specific groups of women with clinically symptomatic UFs, but we need more studies which would examine its safety and efficacy [126]. No current RCTs about the use of cabergoline in UF management are available.

### 3.7. Others

We mentioned the top six current alternative agents in UF therapy. There are also other substances which might have an impact in this field. Unfortunately, their trial data and available evidence are limited. We will attempt to briefly explain each of these substances.

Combined oral contraception (COC) has a well-documented efficacy in case of excessive uterine bleeding [129]. The bleeding often accompanies UFs and is in fact UF-dependent [130]. COC is a common first-choice therapy among the gynecologists in women with such complaints. The results of the ESHRE (European Society of Human Reproduction and Embryology) group demonstrated that the use of COC before the age of 17 might have a weak association with UF occurrence [129]. According to a study by Qin et al., COC does not have a significant influence on UF tumor growth and occurrence [131]. Some clinical trials have confirmed that COCs containing estradiol valerate and dienogest are in fact effective in treating excessive bleeding. However, they investigated patients without organic pathologies such as UFs [132]. Current data on the efficacy of COC in UF-therapy are limited.

Gestrinone is a synthetic steroid with mixed progestogenic and antiprogestogenic effects, with some androgenic and antiestrogenic activity, used in gynecology [133]. It has an inhibitory effect on the pituitary gland and is comparable to danazol in its function [134]. According to Zhu et al., it inhibits growth of UFs via its antagonistic effect on the estrogen and progesterone receptors [36]. In various studies by Coutinho et al., administration of gestrinone reduced UF tumor volume and uterine bleeding [135,136,137]. The side effects of gestrinone were as follows: weight gain and androgenic features such as decreased breast size, acne, seborrhea, and hirsutism [136]. In light of rather limited beneficial effects of using gestrinone in UF therapy, no RCTs comparing gestrinone and other agents or even a placebo are currently available [114].

Curcumin is a yellow substance produced by some plants. It is used as food seasoning, cosmetic ingredient, or herbal supplement. Curcumin is a diarylheptanoid belonging to the curcuminoid family (natural phenols), which has anti-inflammatory, antioxidative [138], and anti-cancer activities, including the inhibition of initiation, progression, invasion, and metastasis formation [139]. According to the available data, curcumin might be applicable in therapy but its low bioavailability and solubility, as well as its rapid metabolism create a challenge [139]. A study by Shishodia et al., revealed that curcumin might inhibit proliferation and fibrosis and regulate apoptosis in mantle cell lymphoma [90,140]. In a study by Malik et al., curcumin demonstrated an inhibitory effect on UF cell proliferation and production of ECM [141]. This inhibitory effect on UF cells was also described by Tsuiji et al., who presented data that curcumin suppresses UF cell proliferation via the activation of PPARγ and decreased proteoglycan expression in ECM [142]. In our opinion, the available data do not support the wider use of curcumin in UF therapy and additional evidence is needed.

SB525334 is a potent and selective inhibitor of TGF-β receptor I (ALK5) [143]. This agent has been found to prevent pulmonary and renal fibrosis [143]. The TGF-β pathway is one of the most important pathways in UF biology [1]. Some authors believe that SB525334 might be a new high-quality treatment in UF therapy [108]. According to a study by Laping et al., treatment with this agent decreases the incidence, number, and size of UF tumors in a mutant rodent model [144]. Due to incomplete data, further studies are necessary to prove that SB525334 might be useful in UF therapy.

Various literature reports also demonstrated that non-steroidal anti-inflammatory drugs (NSAIDs) can be effective in reducing heavy menstrual bleeding, due to their influence on the endometrial cyclooxygenase levels [114,145]. These drugs were widely tested in abnormal uterine bleeding, but there is no sufficient data to recommend them in women with UFs and other symptoms as they do not exert any influence on fibroid volume [114]. Very recently, Gao et al. published their data about the effect of acetylsalicylic acid on UF cells. Their results suggested that aspirin inhibits UF cell growth by the regulation of K-Ras pathways [146]. While interesting, nothing else is known in this area. We hope that we will receive more data about the potential usefulness of aspirin in the prophylaxis or UF therapy very soon. 

One of the last substances of note is pirfenidone, a pyridine molecule used as an antifibrotic agent [147,148] in the treatment of pulmonary fibrosis [148]. Studies about pirfenidone and UFs demonstrated that pirfenidone was an effective inhibitor of myometrial and UF cell proliferation, and that it reduced ECM mRNA levels [147]. We cannot recommend it as a standard treatment due to lack of published data.

Finally, there are several studies regarding the potential role of Ro 41-0960, a synthetic COMT inhibitor [149] and tocopherol analogs in the treatment of UFs [150] but these agents need more evidence.

## 4. Conclusions

UFs constitute a serious health problem for many women of reproductive age, as well as those approaching, or actively in, menopause. Prophylaxis of UFs is practically non-existent, while treatment is often costly and expensive. Surgery is standard in symptomatic UF treatment. UPA has been available for several years. Other forms of treatment are not accepted by patients. In the case of more effective agents, the main problem is the chronic use of drugs and the related side effects. In the case of other agents, their poor effectiveness remains the greatest issue. In our opinion, additional solutions are necessary to create appropriate schemes of pharmacological treatment for different groups of women (obese or non-obese, Caucasian or African-American, pre- or post-menopausal, etc.).

Early prevention and treatment of UFs in women from high-risk groups should be our priority. Innovative forms of UF management are under intensive investigation and may be promising options in the near future. There are several studies about the role of the abovementioned agents in UF prophylaxis and therapy in humans. Many of them evaluated vitamin D, paricalcitol, EGCG, elagolix, AIs, and cabergoline and deemed them safe and effective. The next step in such projects should be properly constructed RCTs, carried out by successive phases. However, the agents on the current list had only one registered trial [101], or none at all. In the case of further positive observations, these agents could become the new generation of drugs in the treatment of UFs, even in the era of UPA.

Perhaps the future solution will be to identify high-risk groups before the onset of UFs and implement preventive methods on the basis of the presented literature. Vitamin D and green tea extract might be optimal in such cases. Recent attempts to create a new, cheap, safe, and effective drug for the treatment of UFs remain in the very early stages, and their success has not been yet determined. Recent findings suggest that substances such as paricalcitol and elagolix may be the formulas for the future as they have minimal or moderate side effects and high levels of efficacy in UF therapy.

## Abbreviations

25(OH)D	25-hydroxyvitamin D
AI	aromatase inhibitor
ALK5	transforming growth factor-β type 1 receptor
BCL	B-cell lymphoma
CDK	cyclin-dependent kinase
COMT	catechol-*O*-methyltransferase
ECG	epicatechin gallate
EGC	epigallocatechin
EGCG	epigallocatechin gallate
ER	estrogen receptor
GnRH	gonadotropin-releasing hormone
MMP	matrix metalloproteinase
mRNA	messenger RNA
PCNA	proliferating cell nuclear antigen
PPAR	peroxisome proliferator-activated receptor
PR	progesterone receptor
QoL	quality of life
RCT	randomized control trial
SPRM	selective progesterone receptor modulator
TGF-β	transforming growth factor beta
TIMP	tissue inhibitor of metalloproteinase
UF	uterine fibroid
UPA	ulipristal acetate
VDR	vitamin D receptor
